# Association between urinary metals and leukocyte telomere length involving an artificial neural network prediction: Findings based on NHANES 1999–2002

**DOI:** 10.3389/fpubh.2022.963138

**Published:** 2022-09-12

**Authors:** Fang Xia, Qingwen Li, Xin Luo, Jinyi Wu

**Affiliations:** Department of Public Health, Wuhan Fourth Hospital, Wuhan, China

**Keywords:** leukocytes telomere length, urinary metals, NHANES, ANN, aging people

## Abstract

**Objective:**

Leukocytes telomere length (LTL) was reported to be associated with cellular aging and aging related disease. Urine metal also might accelerate the development of aging related disease. We aimed to analyze the association between LTL and urinary metals.

**Methods:**

In this research, we screened all cycles of National Health and Nutrition Examination Survey (NHANES) dataset, and download the eligible dataset in NHANES 1999–2002 containing demographic, disease history, eight urine metal, and LTL. The analysis in this research had three steps including baseline difference comparison, multiple linear regression (MLR) for hazardous urine metals, and artificial neural network (ANN, based on Tensorflow framework) to make LTL prediction.

**Results:**

The MLR results showed that urinary cadmium (Cd) was negatively correlated with LTL in the USA population [third quantile: −9.36, 95% confidential interval (CI) = (−19.7, −2.32)], and in the elderly urinary molybdenum (Mo) was positively associated with LTL [third quantile: 24.37, 95%CI = (5.42, 63.55)]. An ANN model was constructed, which had 24 neurons, 0.375 exit rate in the first layer, 15 neurons with 0.53 exit rate in the second layer, and 7 neurons with 0.86 exit rate in the third layer. The squared error loss (LOSS) and mean absolute error (MAE) in the ANN model were 0.054 and 0.181, respectively, which showed a low error rate.

**Conclusion:**

In conclusion, in adults especially the elderly, the relationships between urinary Cd and Mo might be worthy of further research. An accurate prediction model based on ANN could be further analyzed.

## Introduction

As far as we know, toxic metals had cumulative biological effects in human, and essential metals supported normal physiological body functions. However, metal binding proteins lack specificity, which were responsible for absorption and transportation of essential metals and, final control of their homeostasis (1). These metallothioneins could conduct molecular simulations so that nutritious essential metal could be replaced by toxic metal. Deficiency or excess of essential metals would cause damage to biological processes, and non-essential metals might have toxic effects (2). Toxic metals entered the human body in many ways, such as food, drinking water, and air. It was reported that exposure to heavy metals would result in varying degrees of lipid peroxidation, DNA damage, and protein modification. Epidemiological surveys suggested that accumulated heavy metals lead to damage of organs, and further resulted in chronic kidney disease, neurological development disorders, cardiovascular disease (CVD), neuronal damage, diabetes, and cancer (3, 4).

Telomeres were DNA protein complexes that protected the ends of eukaryotic chromosomes. Telomeres shortened each time a cell divided, partly because the ends of telomeric DNA could not replicate. Oxidative stress promoted telomere shortening, while telomerase could prolong telomere. However, the expression of telomerase was low in most human cells, making telomeres vulnerable to oxidative stress (5). When telomeres were severely shortened, cell senescence was triggered. Cell senescence led to functional defects and the secretion of inflammatory factors. Telomere shortening was not only a key mechanism of cell senescence, but also contribute to body's senescence. In epidemiological studies, researchers reported that the shortening of leukocytes telomere length (LTL) was associated with aging and several diseases including CVD, type 2 diabetes, dementia, and cancer. Although a twin study showed that the LTL was partly heritable, the heritability of twins decreased with age growth and lead to their differences in LTL, indicating that environmental factors played a role in LTL (6). Leukocytes telomere length was also associated with behavioral risk factors, such as smoking, alcohol, and socioeconomic status.

Chronic diseases and tumors caused by toxic heavy metals were consistent with LTL-related diseases. Telomere attrition might be an important mechanism of metal accelerating telomere shortening. Harmful heavy metals aggravated oxidative stress and cytokines production (7). Therefore, analyzing the relationship between heavy metals and LTL was an important direction to elaborate the mechanism of heavy metals and diseases. Urinary heavy metals were excreted by the body after absorbing, which could reflect the metal accumulated in kidney and other tissues (8). Therefore, this analysis would focus on the relationship between urinary heavy metals and LTL.

Deep learning was an important part of artificial intelligence. It became the main solution in image recognition, language recognition, and natural language processing (9). Deep learning algorithm was based on the traditional algorithm, the deep convolution neural network was invented to meet the requirements of feature extraction and learning with a big data (10, 11). In terms of hardware configuration, people proposed distributed computing and cloud computing to meet the high requirements for training environment, so that more people can participate in the research of deep learning. Many of the world's top high-tech companies set up laboratories to find a more convenient and rapid development mode for deep learning (12, 13). Tensorflow was a symbolic mathematical system, which was originally developed by Google brain group for deep neural networks in mechanical learning. It was an open-source software platform that uses data flow graphs to calculate numerical values (10–13). Tensorflow was a complete toolkit that could realize the training, testing, parameter adjustment and prediction of convolutional neural networks, and make the modularization deep learning. The principle of modularization made it easy to modify and expand the model network layer and loss function. Consequently, we made the prediction of LTL based on Tensorflow framework.

Previous studies focused on the risk factors exploration on LTL with limited methods to make prediction. We firstly screened the possible hazardous urinary metal for LTL. Since the prediction of LTL was also important, we tried to use the popular ANN platform Tensorflow.

## Methods

### Dataset

National Health and Nutrition Examination Survey (NHANES) was a nationally representative cross-sectional survey of the nutritional and health status of non-hospitalized civilians in the United States, conducted annually by the National Centers for Health Statistics (NCHS) and the Centers for Disease Control and Prevention (CDC). All the data could be obtained in the official website of American Centers for Disease Control and Prevention (https://www.cdc.gov/nchs/nhanes). In this research, the NHANES in 1999–2002 containing demographic, disease history, urine metal, and leukocyte telomere length (LTL) was achieved.

The demographic data included age, gender, race, education level, marital status, alcohol, smoking, body mass index (BMI), poverty income ratio (PIR), diabetes mellitus, and hypertension. Urine metal contained barium (Ba), cadmium (Cd), cobalt (Co), cesium (Cs), molybdenum (Mo), lead (Pb), antimony (Sb), and thallium (Tl). The outcome of LTL was skew continuous variable.

**Figure 1:**
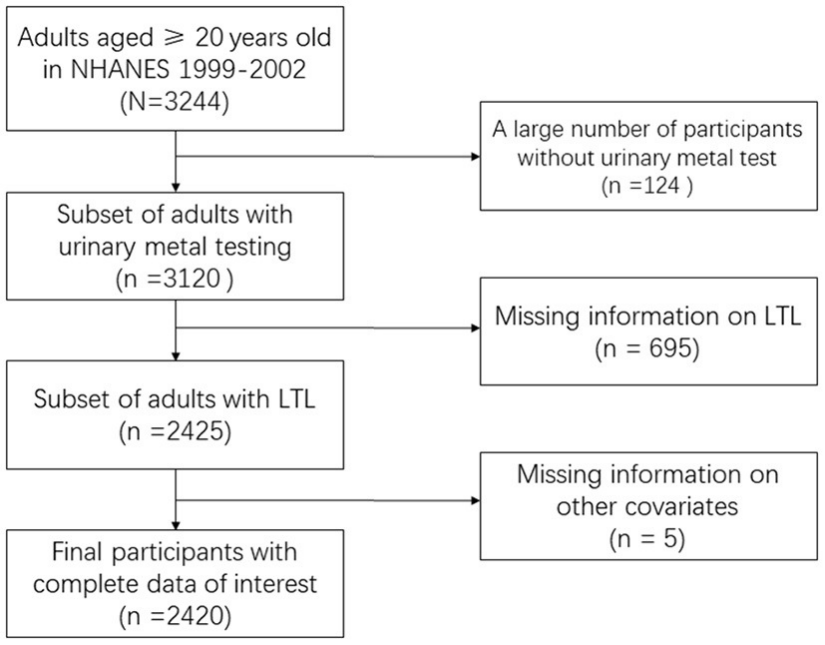
Flowchart of dataset selection.

To screen LTL and related datasets, we searched related data in NHANES 1999–2002. In the raw data, there were 5,352 participants, 5,157 respondents having lab data, and 2,555 with OA status data. Finally, 2,420 participants having demographic, disease history, eight urine metal, and outcome of LTL were included (Figure 1).

### Evaluation of LTL

From description of LTL detection in NHANES website, the telomere length test was carried out in Dr. Elizabeth Blackburn at the University of California, San Francisco, using the quantitative polymerase chain reaction (PCR) method to measure telomere length relative to standard reference DNA (T/S ratio) (14). Each sample was tested three times in three different days. Samples were tested in duplicate wells, resulting in six data points. The sample plates were tested in groups of three plates. Each test plate contained 96 control wells with eight control DNA samples. Tests with eight or more invalid control wells were excluded from further analysis. The control DNA values were used to normalize the variability between the series. Executions with more than four control DNA values less than 2.5 standard deviations from the mean of all test were excluded from the additional analysis. For each sample, any potential outliers were identified and excluded from the calculations. The mean and standard deviation of the T/S ratio were then calculated normally.

### Assessment of urine metals

Urinary metal was used as an exposure assessment in this research, because it was a substitute for cumulative exposure and reflected the metal accumulated in the kidney and other tissues. Inductively coupled plasma mass spectrometry (ICP-MS) was a multi-element analysis technology (15). The liquid sample was introduced into the ICP through a nebulizer and a spray chamber carried by a flowing argon stream. By coupling RF power to flowing argon, a plasma was produced, in which the main components were positive argon ions and electrons. The sample passed through a plasma region with a temperature of 6,000–8,000 K. Heat atomized the sample and then ionized the atoms. Ions and argon entered the mass spectrometer through the interface, which separated the ICP working at atmospheric pressure from the mass spectrometer working at 10^−6^ Torr pressure. The mass spectrometer allowed the detection of ions in a rapid sequence to determine the individual isotopes of the elements. The electrical signal generated by the ion detection processed into digital information to indicate the strength of the ion and the subsequent element concentration. Seven elements in urine, including barium (Ba), cobalt (Co), cesium (Cs), molybdenum (Mo), lead (Pb), antimony (Sb), and thallium (Tl), were measured by ICP-MS according to the method of Mulligan et al. Urine samples were diluted 1 + 9 with 2% v/V double distilled concentrated nitric acid (GFS chemicals Inc., Columbus, OH), which contained iridium and rhodium for multiple internal standardization. In addition, Urine cadmium (Cd) levels were corrected for interference from molybdenum oxide. Corrected cadmium levels = original value for the cadmium – [(0.00175^*^ Molybdenum) – 0.0136].

### Covariates

Information on demography and disease history was collected by questionnaires. Demographic data were age (continuous), BMI (continuous), PIR (continuous), gender (male, female), race (non-Hispanic white, non-Hispanic black, other Hispanic, Mexican American, others), education (more than high school, high school or equivalent, and less than school), marital status (married, widowed/divorced/separated, and never married), smoking status (yes and no), alcohol status (yes and no), hypertension (yes and no), diabetes (yes, no, and borderline). The BMI (kg/m^2^) was classified as normal weight <25, overweight 25 to <30, and obesity ≥30. The Department of Health and Human Services' poverty guidelines were used as the poverty measure to calculate PIR. Diabetes was defined as reaching a fasting glucose level of ≥126 mg/dl or reporting a previous diagnosis (16). Hypertension was defined as resting blood pressure persistent at 140/90 mmHg or reporting a previous diagnosis (17).

### Statistical analysis

To analyze the association between LTL and 8 urine metals including barium (Ba), cadmium (Cd), cobalt (Co), cesium (Cs), molybdenum (Mo), lead (Pb), antimony (Sb), and thallium (Tl) in the two groups (whole and aging population ≥60 years old), we conducted a three-step analysis.

We firstly describe the demographic and disease history data in overall and aging groups. Meanwhile, we analyzed the geometric mean (GM), LOD, and four quantiles of eight urine metals. Based on these results, chi-squared test, Cochran-Mantel-Haenszel test, and *t*-test were used to analyze the difference of demographic data in overall and aging population groups. Secondly, we conducted multiple linear regression (MLR) to find association between natural log-transformed LTL and quantiles of urine metals. Moreover, subgroup MLRs adjusted gradually for demographic and disease history were carried out to identify meaningful hazardous urine metals associated with changes of LTL. Thirdly, to make an accurate prediction of LTL, artificial neural network (ANN) was trained. All the variables in MLRs were put into the ANN model and three hidden layers were established to make a reliable prediction. All the analysis above were conducted in R software 4.1.2 (The R Foundation for Statistical Computing, USA). Two-sided *P* < 0.05 was considered statistically significant.

### Artificial neural network prediction

In this research, an ANN framework: Tensorflow (developed by Google brain team based on the idea of a dataflow graph for building models) was adapted. Artificial neural network had three main components: (1) A group of synapses or connections was characterized by “weight,” in which the input signal was connected to the neuron through connection weight; (2) An adder would add all weighted signal contributions; (3) The activation function (transfer function) affected neurons, which limited the amplitude of the network output and provided a permissible range for the output signal of finite value (18, 19). The common activation functions included linear, quadratic, geometric, logical, and rectified linear unit (ReLU).

The input layer and dense hidden layer used the activation function ReLU, which included specifying the value of neurons <0 as 0, and respecting the value of neurons ≥0 when its value is 0, as shown in the equation


$$ReLU(x)=\{\begin{array}{c} x,\;x\geq 0\\ 0,\;x<0 \end{array}$$

Finally, in order to verify the results of ANN, three indicators were generated, including LOSS function (Squared error loss), MAE (mean absolute error), and scatter plot of actual and predicted values. Artificial neural network was mainly trained by reducing the iterations of LOSS function using gradient method. The commonly used techniques for calculating the LOSS function included mean square error, MAE, binary cross entropy, and Poisson.

In this study, after analyzing the relationship between urinary metal and telomere length, we carried out further prediction analysis. Artificial neural network became a popular prediction algorithm in recent years. In this study, “keras” (a high-level ANN application programming interface written in python) and “neuralnet” package (flexible ANN training program) were used to deploy the “Tensorflow” framework, setting hidden layers, and calculating with logistic function in each layer. The data was divided into training set and test set according to 8:2, 2,420 participants were divided into 1,936 in the training set and 404 in test set. Then the data sets were normalized, respectively. Firstly, the preliminary model was constructed, 25 variables were input (demographic, behavioral, disease, and urinary metal data included in this study), one hidden layer with five neurons and one output result were set, and the model compilation indicators were reported including LOSS function and MAE. Then we carried out model fitting and set the number of iterations to 100. Finally, we put the test set data into the trained model for prediction and verification. Based on the above steps, we also optimized the model parameters to reconstructed the model, changing one hidden layer to three hidden layers (100 neurons and 0.76 dropout rate in the first layer, 15 neurons and 0.5 dropout rate in the second layer, and 7 neurons and 0.2 dropout rate in the third layer). All layers adopted ReLU, and finally got an output. The whole process was conducted using R 4.1.2 (The R Foundation for Statistical Computing, USA).

## Results

### Characteristics of participants

The characteristic distribution of the study population (*n* = 2,420) in the total sample and aging sample (*n* = 821) was shown in Table 1. In overall group, the sample was mainly composed of the middle aged (49.5 ± 18.7), middle income (PIR 2.66 ± 1.64), overweight (37.36%), female (51.53%), White, Non-Hispanic (50%), more than high School (41.78%), married (59.01%), alcoholic (67.4%), non-smoking (50.58%), non-hypertension (70.41%), and non-diabetes (88.47%). In aging group, the main compositions included the elderly (71.4 ± 7.93), middle income (PIR 2.63 ± 1.57), overweight (42.51%), male (53.23%), Non-Hispanic White (56.64%), less than high school (42.27%), married (64.19%), alcoholic (61.63%), smokers (52.86%), non-hypertension (50.91%), and non-diabetes (79.42%). Between overall and aging groups, several characteristics had significant difference including BMI distribution, gender, race, education, marital status, alcohol, hypertension, and diabetes.

**Table 1:** Comparison between overall and aging population in weighted characteristics of the NHANES 1999–2002.

	**Overall (*N* = 2,420)**	**Weighted**	**Aging (*N* = 821)**	**Weighted**	***t* or chi-squared value**
**Age**
	49.5 ± 18.7		71.4 ± 7.93		−46.58^*^
**PIR**
	2.66 ± 1.64		2.63 ± 1.57		0.47
**BMI**
	747	30.87%	226	27.53%	7.23^*^
	904	37.36%	349	42.51%	
	769	31.78%	245	29.84%	
**Gender**
Male	1,173	48.47%	437	53.23%	5.36^*^
Female	1,247	51.53%	384	46.77%	
**Ethnicity**
Mexican American	609	25.17%	182	22.17%	12.85^*^
Other Hispanic	122	5.04%	31	3.78%	
White, Non-Hispanic	1,210	50.00%	465	56.64%	
Black, Non-Hispanic	409	16.90%	128	15.59%	
Other	70	2.89%	15	1.83%	
**Education**
Less than high school	815	33.68%	347	42.27%	24.88^*^
High school diploma	594	24.55%	204	24.85%	
More than high school	1,011	41.78%	270	32.89%	
**Marital status**
Married	1,428	59.01%	527	64.19%	228.26^*^
Widowed	208	8.60%	187	22.78%	
Divorced	207	8.55%	66	8.04%	
Separated	79	3.26%	17	2.07%	
Never married	350	14.46%	14	1.71%	
Living with partner	144	5.95%	8	0.97%	
**Alcohol**
Yes	1,631	67.40%	506	61.63%	8.67^*^
No	788	32.56%	314	38.25%	
**Smoking**	
Yes	1,189	49.13%	434	52.86%	3.35
No	1,224	50.58%	384	46.77%	
**Hypertension**
Yes	714	29.50%	403	49.09%	102.9^*^
No	1,704	70.41%	418	50.91%	
**Diabetes**
Yes	238	9.83%	146	17.78%	42.32^*^
No	2,141	88.47%	652	79.42%	
Borderline	41	1.69%	23	2.80%	

* *p* < 0.05.

### Distribution of urine metals

Table 2 showed the limit of detection (LOD), GM, 95% confidential interval (CI), and four quantiles of the eight urine metals among total and aging participants in our study. All eight urinary metals were recorded using ng/ml. Besides, we analyzed the correlation among all the variables in the regression model. Supplementary Figure 1 showed that all urine metals and creatinine had some correlations.

**Table 2:** Urine metals (ng/ml) in overall and aging population of the NHANES 1999–2002.

**Overall (*N* = 2420)**	**≥LOD (%)**	**GM (95%CI)**	**Quartile 1**	**Quartile 2**	**Quartile 3**	**Quartile 4**
LTL		0.99(0.99, 1.01)	≤ 0.84	0.84–0.99	0.99–1.17	>1.17
Barium	0.084	1.29(1.24, 1.35)	≤ 0.66	0.66–1.39	1.39–2.65	>2.65
Cadmium	0.055	0.31(0.3, 0.33)	≤ 0.17	0.17–0.32	0.32–0.62	>0.62
Cobalt	0.024	0.36(0.35, 0.37)	≤ 0.22	0.22–0.38	0.38–0.59	>0.59
Cesium	0.13	4.46(4.33, 4.6)	≤ 3.01	3.01–4.96	4.96–7.26	>7.26
Molybdenum	0.8	42.46(40.97, 44)	≤ 25.4	25.4–47.4	47.4–77.2	>77.2
Lead	0.03	0.82(0.79, 0.85)	≤ 0.5	0.5–0.9	0.9–1.5	>1.5
Antimony	0.022	0.13(0.12, 0.13)	≤ 0.09	0.09–0.13	0.13–0.19	>0.19
Thallium	0.018	0.16(0.15, 0.16)	≤ 0.11	0.11–0.18	0.18–0.26	>0.26
Aging (*N* = 821)	**≥LOD (%)**	**GM (95%CI)**	**Quartile 1**	**Quartile 2**	**Quartile 3**	**Quartile 4**
LTL		0.88(0.87, 0.90)	≤ 0.75	0.75–0.88	0.88–1.02	>1.02
Barium	0.084	1.1(1.02, 1.18)	≤ 0.59	0.59–1.1	1.1–2.2	>2.2
Cadmium	0.055	0.41(0.38, 0.43)	≤ 0.24	0.24–0.44	0.44–0.75	>0.75
Cobalt	0.024	0.31(0.29, 0.33)	≤ 0.2	0.2–0.32	0.32–0.5	>0.5
Cesium	0.13	4.16(3.97, 4.37)	≤ 2.9	2.9–4.55	4.55–6.65	>6.65
Molybdenum	0.8	39.17(36.85, 41.63)	≤ 23.2	23.2–41.1	41.1–71.1	>71.1
Lead	0.03	0.87(0.82, 0.93)	≤ 0.5	0.5–0.9	0.9–1.6	>1.6
Antimony	0.022	0.11(0.11, 0.12)	≤ 0.08	0.08–0.11	0.11–0.16	>0.16
Thallium	0.018	0.14(0.13, 0.14)	≤ 0.09	0.09–0.14	0.14–0.22	>0.22

### Associations of urine metal metabolites with LTL

In this section, we fitted MLR in steps to validate the stability of result. The dependent variable LTL was log transformed and normalized, but the normality analysis showed that all variables did not obey normality. In Model 3 of overall group included all covariates and only Cd was found significant in association with shortening LTL (third quantile: −9.36, 95%CI = [−19.7, −2.32)]. In aging group, Mo was associated with prolonging LTL in model 3 [third quantile: 24.37, 95%CI = (5.42, 63.55)] (Table 3).

**Table 3:** Percent difference (95% CI) in leukocyte telomere length (T/S ratio) by urine metals (ng/mL) in overall and aging population of the NHANES 1999–2002.

	**Overall**			**Aging**		
	**Model 1**	**Model 2**	**Model 3**	**Model 1**	**Model 2**	**Model 3**
**Urinary barium**						
Quartile 1	Reference	Reference	Reference	Reference	Reference	Reference
Quartile 2	−0.32 (−10.8, 11.4)	−1.85 (−11.42, 8.75)	−1.67 (−11.3, 8.99)	7.36 (−12.7, 32.02)	8.33 (−11.46, 32.55)	4.46 (−10.58, 22.04)
Quartile 3	1.79 (−9.35, 14.31)	3.32 (−7.1, 14.91)	3.37 (−7.05, 14.97)	0.93 (−15.46, 20.49)	1.19 (−14.47, 19.71)	2.97 (−17.83, 29.05)
Quartile 4	0.02 (−3.51, 3.68)	−0.06 (−3.26, 3.25)	−0.17 (−3.38, 3.15)	−0.89 (−6.1, 4.61)	−1.88 (−6.92, 3.45)	−2.76 (−8.23, 3.03)
p trend	0.74	0.17	0.23	0.62	0.65	0.67
**Urinary cadmium**						
Quartile 1	Reference	Reference	Reference	Reference	Reference	Reference
Quartile 2	−7.51 (−18.16, 4.52)	−3.55 (−14.27, 8.52)	−3.46 (−14.24, 8.67)	−10.67 (−25.92, 7.72)	−8.88 (−24.09, 9.37)	1.41 (−18.43, 26.08)
Quartile 3	**−18.47 (−27.89**, −7.83)*	**−8.99 (−19.38**, −2.73)*	**−9.36 (−19.7**, −2.32)*	−5.6 (−24.64, 18.26)	−9.48 (28.1, 13.97)	−5.67 (−21.95, 14)
Quartile 4	**−4.09 (−8.59**, −0.63)*	**3.05 (−1.52**, −7.83)*	2.68 (−1.93, 7.5)	1.94 (−6.96, 11.68)	5.25 (−3.92, 15.29)	0.74 (−6.76, 8.85)
p trend	<0.0001*	0.4	0.31	0.01*	0.03*	0.04*
**Urinary cobalt**						
Quartile 1	Reference	Reference	Reference	Reference	Reference	Reference
Quartile 2	7.37 (−6.42, 23.19)	8.56 (−4.43, 23.3)*	8.61 (−4.39, 23.39)	2.08 (−22.4, 34.29)	−3.21 (−26.21, 26.96)	6.53 (−13.02, 30.48)
Quartile 3	−1.53 (−17.24, 17.16)	−6.87 (−20.57, 9.18)	−7.97 (−21.49, 7.88)	−8.46 (−30.3, 20.23)	−10.54 (−31.89, 17.5)	−15.5 (−37.78, 14.76)
Quartile 4	2.4 (−2.44, 7.48)	−0.9 (−5.13, 3.53)	−0.87 (−5.11, 3.55)	−6.35 (−12.69, 0.44)*	−3.65 (10.1, 3.27)	−4.17 (−11.59, 3.88)
p trend	0.29	0.89	0.83	0.15	0.6	0.66
**Urinary cesium**						
Quartile 1	Reference	Reference	Reference	Reference	Reference	Reference
Quartile 2	−0.91 (−17, 18.3)	−0.03 (−14.94, 17.49)	0.33 (−14.67, 17.96)	−13.33 (−34.44, 14.57)	−9.24 (−30.95, 13.92)	−3.71 (−24.93, 23.51)
Quartile 3	−9.47 (−25.13, 9.48)	−8.72 (−23.54, 8.98)	−10.07 (−24.71, 7.43)	1.64 (−26.39, 40.33)	−6.55 (−32.05, 28.52)	−8.01 (−34.48, 29.16)
Quartile 4	−1.91 (−7.95, 4.53)	3.5 (2.17, 8.86)*	−3.87 (−9.21, 1.78)	−6.16 (−18.7, 8.31)	−7.16 (−19.4, 6.95)	−1.25 (−16.15, 16.3)
p trend	0.03*	0.69	0.71	0.38	0.45	0.47
**Urinary molybdenum**						
Quartile 1	Reference	Reference	Reference	Reference	Reference	Reference
Quartile 2	−6.31 (−16.66, 5.33)	7.91 (−2.53, 17.29)*	−5.13 (−14.89, 5.76)	−5.09 (−24.14, 18.74)	−2.05 (−21.65, 22.47)	−1.2 (−17.77, 18.72)
Quartile 3	14.22 (−1.89, 32.98)*	11.02 (−3.32, 27.48)*	10.23 (−3.98, 26.55)	3.15 (−17.79, 29.42)	11.51 (−10.97, 39.68)	24.37 (5.42, 63.55)*
Quartile 4	−0.9 (−7.1, 5.71)	2.23 (−3.48, 8.28)	2.09 (−3.61, 8.12)	−0.09 (−9.02, 9.73)	0.17 (−8.53, 9.69)	1.2 (−8.4, 11.8)
	**Model 1**	**Model 2**	**Model 3**	**Model 1**	**Model 2**	**Model 3**
p trend	0.11	0.03*	0.03*	0.08	0.09	0.12
**Urinary lead**						
Quartile 1	Reference	Reference	Reference	Reference	Reference	Reference
Quartile 2	0 (−18.7, 23)	7.23 (−11.67, 30.17)	5.67 (−13.01, 28.36)	1.1 (−30.53, 47.12)	−2.5 (−30.4, 36.57)	−1.58 (−30.13, 38.63)
Quartile 3	−0.28 (−16.76, 19.46)	15.67 (−2.13, 36.7)	15.29 (−2.5, 36.33)	30.57 (−1.02, 72.24)	32.62 (0.5, 75.01)	31.18 (−0.81, 73.49)
Quartile 4	−0.59 (−5.08, 4.1)	2.22 (−2.05, 6.67)	1.97 (−2.31, 6.43)	−0.48 (−7.52, 7.1)	2.71 (−4.71, 10.71)	1.54 (−5.23, 8.79)
p trend	0.0001*	0.12	0.13	0.22	0.73	0.69
**Urinary antimony**						
Quartile 1	Reference	Reference	Reference	Reference	Reference	Reference
Quartile 2	0.67 (−20.16, 26.94)	−3.01 (−20.81, 18.8)	−0.03 (−21.18, 18.5)	−23.31 (−42.59, 2.43)*	−22.4 (−41.52, 2.97)	8.1 (−22.1, 50.01)
Quartile 3	−11.03(−28.76. 11.12)	−14.12(−30.08, 5.48)	−0.15(−30.06, 5.63)	−13.41(−46.52, 40.2)	−18.12(−48.69, 30.67)	−15.3 (−44.6, 29.48)
Quartile 4	0.11(−4.86, 5.35)	0.77(−3.87, 5.64)	0.005(−4.17, 5.34)	1.75(−5.15, 9.15)	1.25(−5.48, 8.46)	2.112 (−5.41, 10.24)
p trend	0.13	0.7	0.71	0.04*	0.04*	0.04*
**Urinary thallium**						
Quartile 1	Reference	Reference	Reference	Reference	Reference	Reference
Quartile 2	1.89 (−14.26, 21.08)	−1.75 (−16.15, 15.13)	−1.3 (−15.82, 15.72)	7.87 (−13.47, 34.48)	7.43 (−13.3, 33.12)	2.58 (−17.4, 27.4)
Quartile 3	13.44 (−14.02, 49.67)	12.85 (−12.7, 45.86)	13.75 (−12.08, 47.16)	6.34 (−20.24, 41.77)	−3.32 (−27.19, 28.38)	−0.58 (−40.94, 67.39)
Quartile 4	1.46 (−6.19, 9.73)	4.16 (−3.42, 12.34)	3.6 (−3.93, 11.72)	−3.59 (13.14, 7.01)	−3.29 (−12.96, 7.46)	−4.3 (−16.76, 10.03)
p trend	0.04*	0.53	0.41	0.04*	0.19	0.16

Bold values were statistically significant with *P* value <0.05. *p < 0.05.

### Artificial neural network for predicting mean T/S ratio

**Figure 2:**
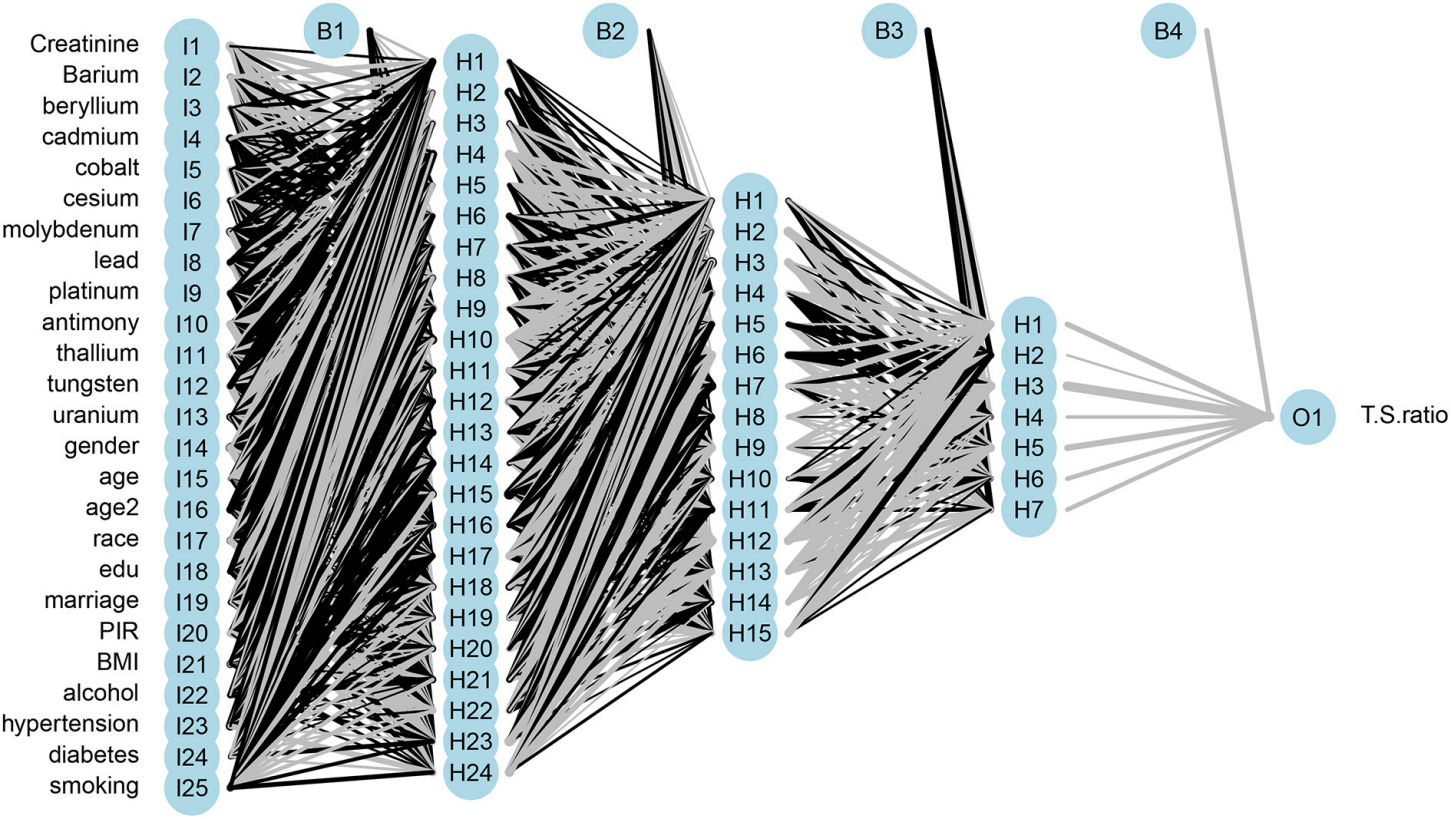
Architecture of multilayer artificial neural network of urinary metal for LTL prediction.

**Figure 3:**
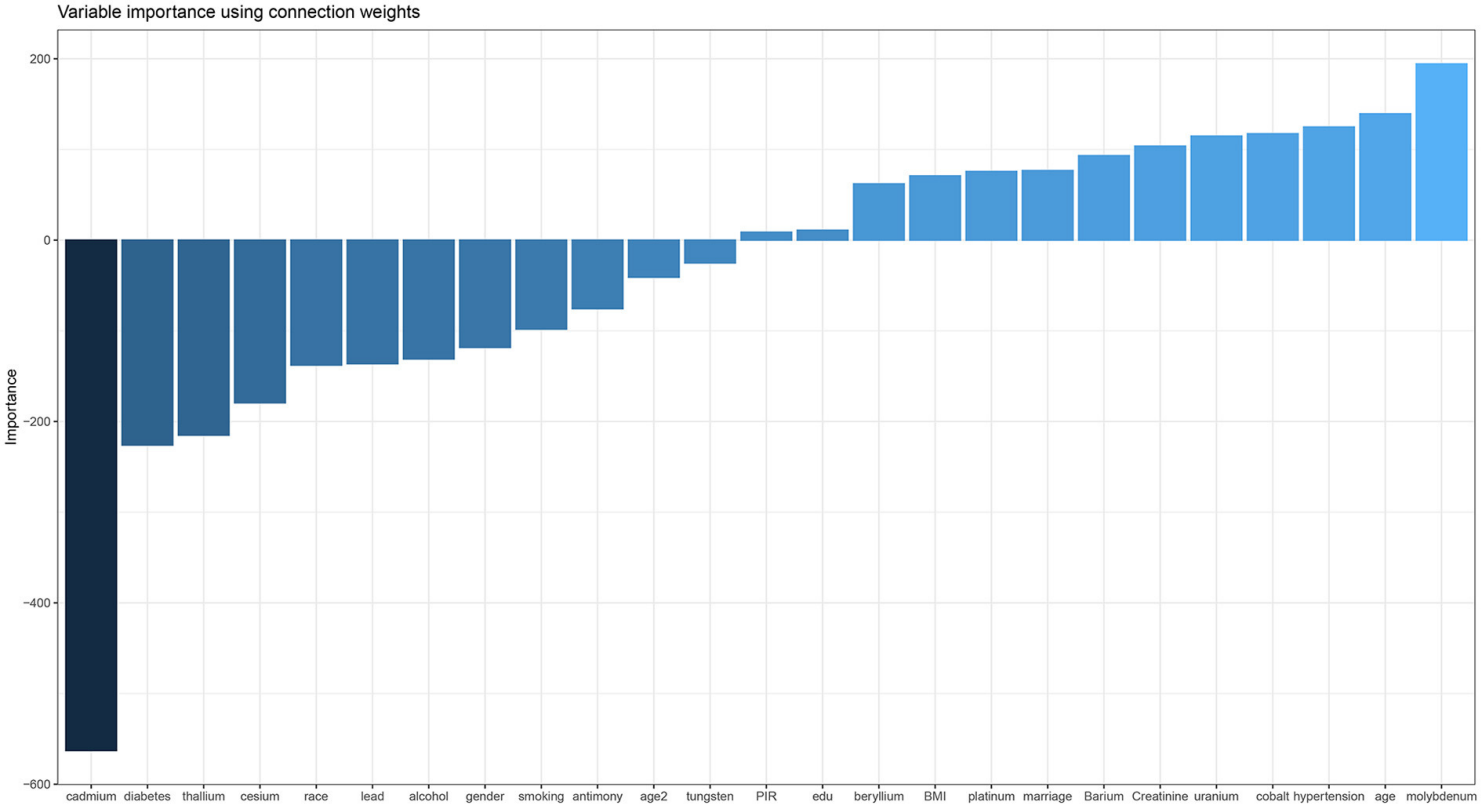
Variable importance plot of ANN.

As shown in Figure 2, based on the risk factors analysis using MLR, we developed an ANN algorithm taking 25 items as the input parameters (data about demography, behavior, disease, and urinary metal). In the hidden layer setting, there were 24 neurons with 0.375 exit rate in the first layer, 15 neurons with 0.53 exit rate in the second layer, and 7 neurons with 0.86 exit rate in the third layer. Logical and rectified linear unit was used in all layers, and finally an output result was obtained. After the model was fitted, we evaluated the importance of the input variables on the model. From Figure 3, it could be seen that the greatest negative impact was urinary cadmium and the greatest positive impact was urinary molybdenum, which were consistent with the significant heavy metals analyzed by MLR.

**Figure 4:**
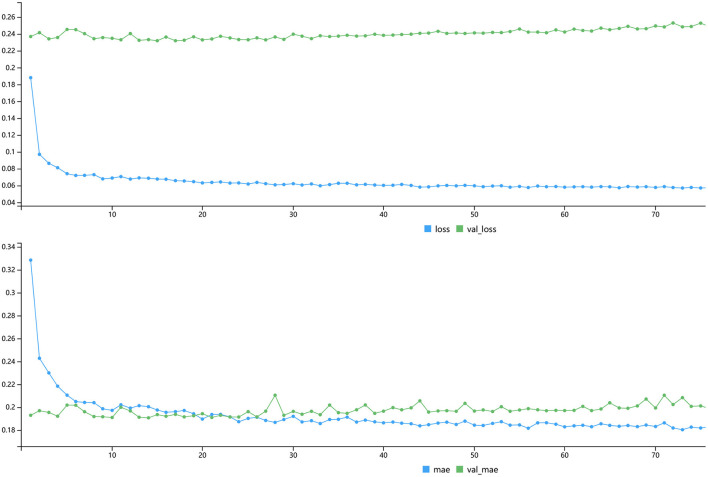
Plot of LOSS and MAE with iterations.

In the process of model fitting, the relationship between the iterations and MAE was conducted in Figure 4. It could be seen that when the iterations reached 10, the minimum values of LOSS and MAE were 0.054 and 0.181, respectively. Since then, MSE would not change with the increase of iteration times, so 10 times iteration was selected as the parameter of model fitting. Finally, we took put test dataset into the model to get the predicted mean T/S ratio. Then scatter plot of predicted value with the original value with trend line was made. Supplementary Figure 2 showed that the scatter points had obvious linearity, indicating that the predicted value was reliable.

## Discussion

The MLR results of the total population showed that there was a negative correlation between urinary Cd and LTL in model 1 (adjusted for urine creatinine), model 2 (adjusted for creatinine and demographic factors), and model 3 (adjusted for creatinine, demographic, and disease factors), indicating the higher the urinary Cd, the shorter the LTL. This result was biologically reasonable because Cd was associated with mechanisms that promoted telomere shortening, including oxidative stress, inhibition of DNA repair, and inflammation. Due to the high level of guanine, telomere was particularly sensitive to oxidative stress. At the same time, telomere might have defects in repairing single strand breaks. Inflammation might accelerate leukocyte telomere shortening by promoting cell renewal, replicative aging, and inducing oxidative stress. Cadmium could stimulate the inflammatory cytokines. Cadmium was a recognized human carcinogen and had been proved to interfere in DNA repair system. Zota et al. analyzed the NHANES 1999–2002 data set and reported the highest quartile of Cd in blood and urine was correlated with shorter LTL, and there was evidence of dose-response relationship (*P* < 0.05) (20).

In the elderly population, model 3 showed a high positive correlation between Mo and LTL [24.37, 95%CI = (5.42, 63.55)]. Domingo Raloso et al. found that the increase of urinary Mo level was related to the increase of redox glutathione ratio (GSSG/GSH), indicating that Mo might reduce the effect of metal oxidative stress. Nakadaira et al. investigated the levels of Mo and Se in sediments and the cancer mortality in 19 areas of Niigata Prefecture, Japan. It was found that Mo could inhibit gastrointestinal cancer (21). Meanwhile, Mo compounds could be used as drugs for detecting and treating tumors. Dhas et al. reviewed MoS2 nanocomposites had attracted extensive attention in the fields of optics, catalysis, electrochemistry, and cancer treatment (22). According to the literature search results, there was little research on association between Mo and LTL, which might be a direction worthy of exploration.

There was no correlation between urinary Co, Cs, Pb, and LTL in overall population and the elderly population, respectively. This was consistent with the research of Zota et al. (20). However, Herlin et al. believe that urinary Pb would affect telomere shortening in children, especially boys (23).

There was no correlation between urinary Sb, Tl, Ba, and LTL in the total population. There was no correlation between urinary Tl, Ba, and LTL in the overall and elderly population. It was reported that ANN could be used for age prediction based on DNA methylation (18). Leukocytes telomere length was closely related to human aging. Perhaps it was meaningful to put urinary metal, demographic data, behavior, and disease history to predict LTL, since the close correlation between LTL and life expectancy. Compared with MLR, ANN especially Tensorflow framework is a brand new technique to make prediction. But, in exploration of risk factors, MLR had good performance which ANN is not good at. Artificial neural network might have better performance than MLR in prediction, since it could include all possible variables and large sample size (10–13).

To our knowledge, this was the first ANN analysis for LTL prediction based on urinary metal. Based on the analysis of the correlations between urinary metal and LTL above in this study, it was suitable for deep learning model. Artificial neural network was a classical algorithm framework of deep learning. It was widely used in categorical variable classifying, continuous variable regression, and time-series data prediction (24–27). Because ANN had the ability to introduce non-linearity in high-dimensional space, large scale factors could be considered to improve the prediction sensitivity and specificity (28, 29).

In the process of model setting, it was found that three hidden layers had good accuracy. The hidden nodes of three layers were reduced from 25 to 24, 24 to 15, and 15 to 7, and the final output result was obtained (Figure 2). This was the best setting after many comparisons in parameters. The training result on the variable importance was also in line with the expectation that Cd had the strongest negative correlation and Mo had the strongest positive correlation (Figure 3). After 10 iterations, LOSS and MAE reach stable minimum values of 0.054 and 0.181, respectively (Figure 4). Finally, the ANN prediction model was obtained. Based on the model, we put the test set into the model and got the scatter diagram of the real value and predicted value. From the diagram, it could be seen that the scatter has good linearity and had a reliable prediction (Supplementary Figure 2).

However, there were several limitations of our search. First, the data used in this study were cross-sectional design, which was impossible to infer the causal relationship between urinary metals and LTL. Secondly, the participants' urine samples were collected and detected at one time, and the single point of metal might not reflect the participants' continuous exposure. Thirdly, although we adjusted some demographic, medical history, and lifestyle factors in linear regression, there were still some confounding variables that affected the results. Fourthly, the lack of information among participants might lead to the exclusion of results. Finally, for the over fitting problem of ANN, it was difficult for us to find the parameters to get high accuracy without over fitting, so we could only choose the relatively best parameters.

## Conclusion

Overall, the main findings in our study were as follows: urinary Cd was negatively correlated with LTL in the total population and urinary Mo was positively correlated with LTL. No correlations were found between urinary Co, Pb, Sb, Cs, Tl, Ba, and LTL. Therefore, in adults especially the elderly, the relationships between urinary Cd, Mo, and LTL might be worthy of further research. In addition, we also constructed an ANN model to make predictions of LTL based on urinary metals, demography, behavior, and disease history, which might help to make prediction of people involving the aging. This could be used in primary prevention of people especially the elderly.

## Data availability statement

The original contributions presented in the study are included in the article/Supplementary material, further inquiries can be directed to the corresponding author/s.

## Author contributions

JW wrote the manuscript and analyzed the date in R language. QL and XL collected the data and screen it. FX designed and reviewed the research. All authors contributed to the article and approved the submitted version.

## Conflict of interest

The authors declare that the research was conducted in the absence of any commercial or financial relationships that could be construed as a potential conflict of interest.

## Publisher's note

All claims expressed in this article are solely those of the authors and do not necessarily represent those of their affiliated organizations, or those of the publisher, the editors and the reviewers. Any product that may be evaluated in this article, or claim that may be made by its manufacturer, is not guaranteed or endorsed by the publisher.

## Supplementary material

Supplementary Figure 1 Correlation matrix of included variables.

Supplementary Figure 2 Scatter plot with line trend of test and prediction value.

## Abbreviations

CVD: cardiovascular disease
LTL: leukocytes telomere length
NHANES: National Health and Nutrition Examination Survey
BMI: body mass index
PIR: poverty income ratio
Ba: barium
Cd: cadmium
Co: cobalt
Cs: cesium
Mo: molybdenum
Pb: lead
Sb: antimony
Tl: thallium
NCHS: National Center for Health Statistics
CDC: Centers for Disease Control and Prevention
PCR: polymerase chain reaction
ICP-MS: Inductively coupled plasma mass spectrometry
GM: geometric mean
CI: confidential interval
LOD: limit of detection
ANN: artificial neural network
ReLU: logical and rectified linear unit
MAE: mean absolute error
MLR: multiple linear regression
GSSG/GSH: redox glutathione ratio
ROS: reactive oxygen species.
