# Supplementary figures and images for: Association between urinary metals and leukocyte telomere length involving an artificial neural network prediction: Findings based on NHANES 1999–2002
Source: Front Public Health. 2022 Sep 12;10:963138. doi: 10.3389/fpubh.2022.963138 (PMC9511050; doi:10.3389/fpubh.2022.963138)

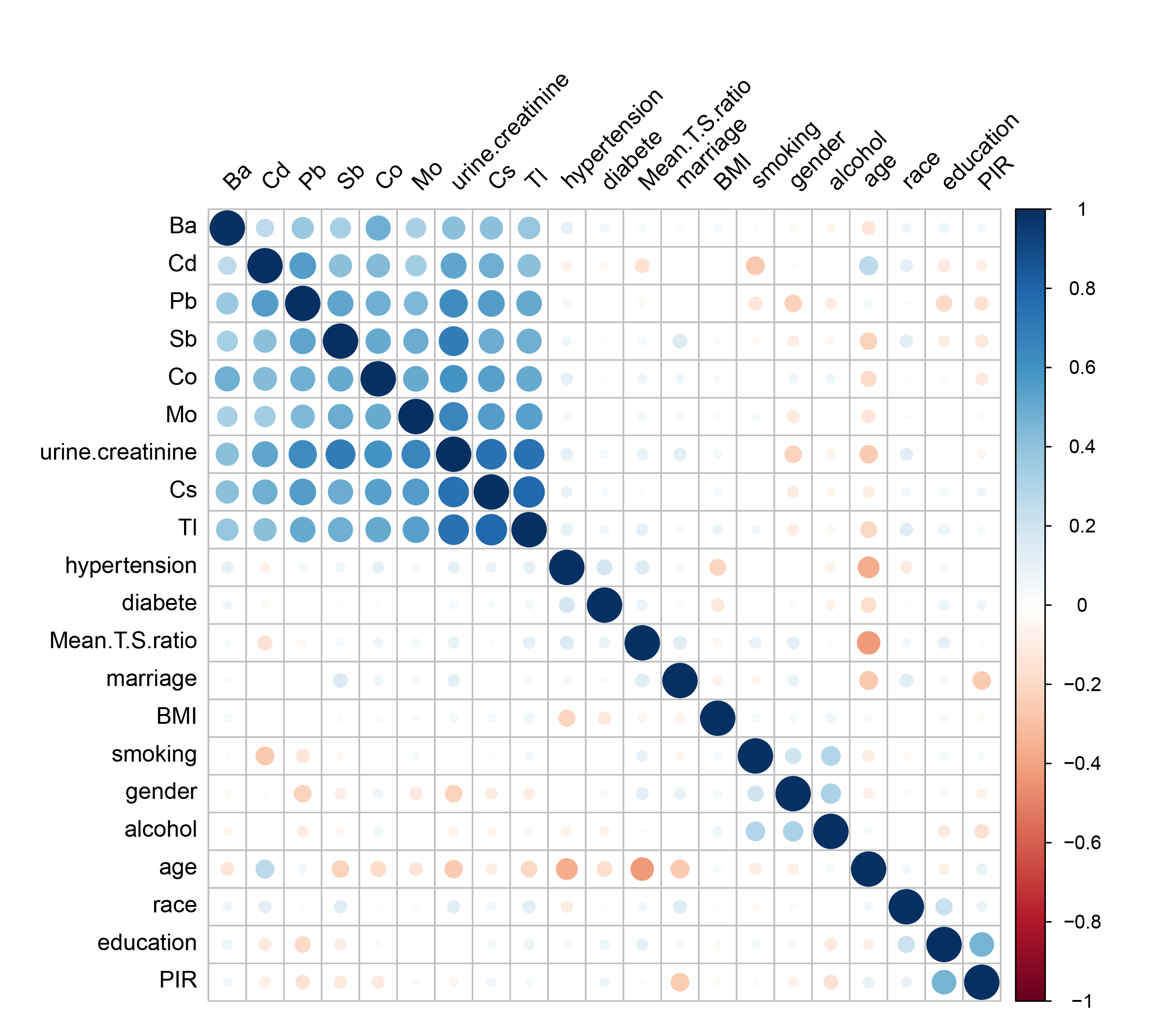

Supplement: Supplementary Figure 1 — Correlation matrix of included variables. [file Image_1.TIF]

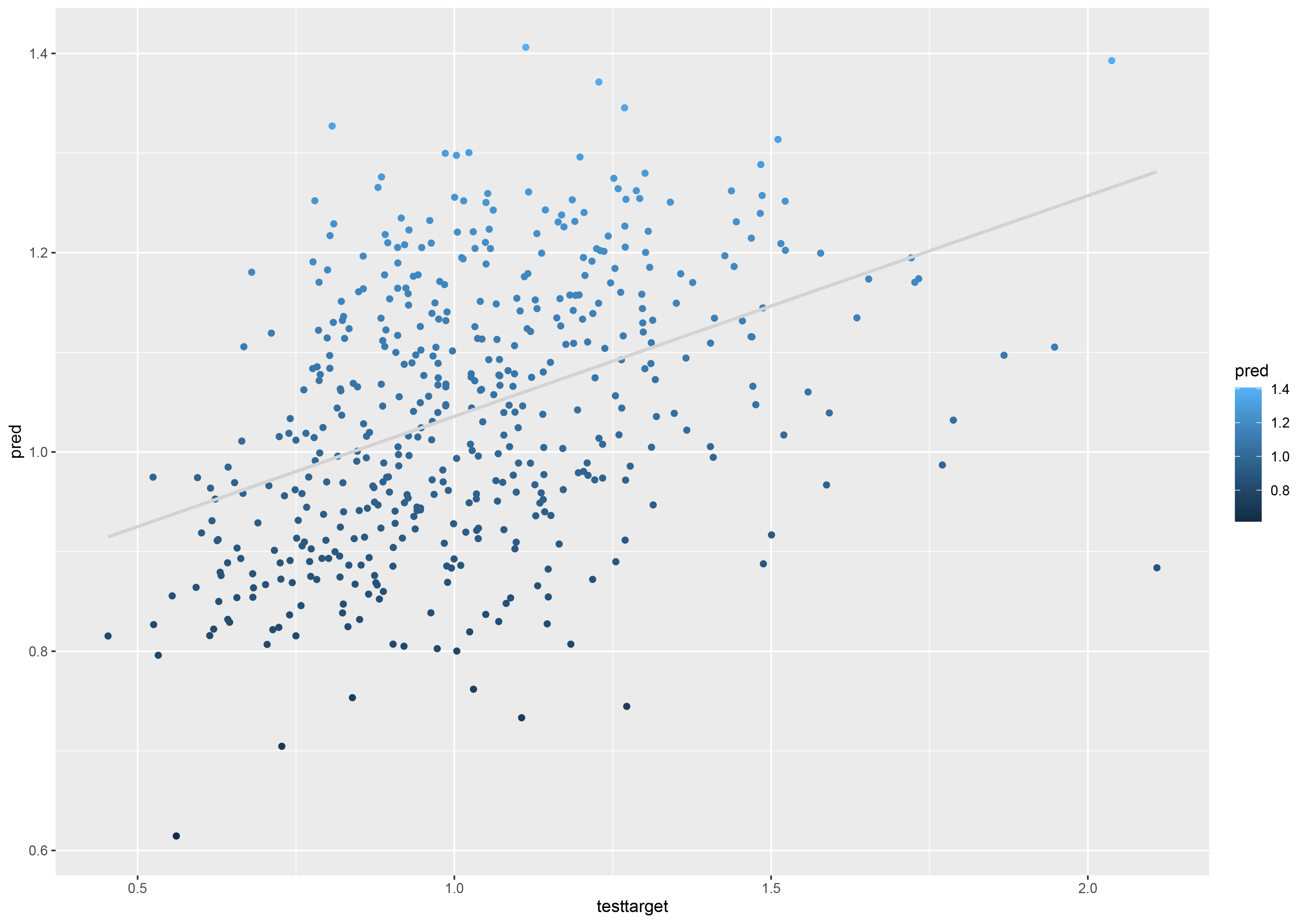

Supplement: Supplementary Figure 2 — Scatter plot with line trend of test and prediction value. [file Image_2.TIF]
